# Immune reconstitution inflammatory syndrome, a controversial burden in the East African context: a systematic review and meta-analysis

**DOI:** 10.3389/fmed.2023.1192086

**Published:** 2023-08-10

**Authors:** Alene Geteneh, Henok Andualem, Demeke Mesfin Belay, Mulugeta Kiros, Sirak Biset

**Affiliations:** ^1^Department of Medical Laboratory Science, College of Health Sciences, Woldia University, Woldia, Ethiopia; ^2^Department of Medical Laboratory Science, College of Medicine and Health Sciences, Debre Tabor University, Debre Tabor, Ethiopia; ^3^Department of Pediatrics and Child Health Nursing, College of Health Sciences, Debre Tabor University, Debre Tabor, Ethiopia; ^4^Department of Medical Laboratory Science, College of Medicine and Health Sciences, Aksum University, Aksum, Ethiopia; ^5^Department of Medical Microbiology, School of Biomedical and Laboratory Sciences, College of Medicine and Health Sciences, University of Gondar, Gondar, Ethiopia

**Keywords:** IRIS, HIV-associated IRIS, HIV, ART, East Africa

## Abstract

**Introduction:**

It is well established that starting antiretroviral therapy (ART) increases a patient's life expectancy among HIV-positive individuals. Considering the HIV pandemic, the major concern is initiation of ARTs to the large segment of HIV infected population, not adverse events from immune restoration. The prevalence of HIV-associated immune reconstitution inflammatory syndrome (IRIS) is poorly estimated due to Africa's underdeveloped infrastructure, particularly in Eastern Africa. Therefore, this study compiled data regarding the magnitude and associated factors of IRIS in the context of Eastern Africa.

**Methods:**

The electronic databases such as Google Scholar, PubMed, Web of Science, and free Google access were searched till 5 June 2021, and the search was lastly updated on 30 June 2022 for studies of interest. The pooled prevalence, and associated factors with a 95% confidence interval were estimated using the random effects model. The I^2^ and Egger's tests were used for heterogeneity and publication bias assessment, respectively.

**Results:**

The development of HIV-associated IRIS in Eastern Africa was estimated to be 18.18% (95% CI 13.30–23.06) in the current review. The two most common predictors of IRIS associated with Eastern Africa were the lower pre-ART CD4 T-cell count of 50 cells/μl and the low baseline body mass index level. Therefore, attention should be focused on the early detection and care of HIV-associated IRIS to reduce the morbidity and death caused by IRIS.

## Introduction

The introduction of antiretroviral therapy (ART) has led to an improvement in the life expectancy of people living with human immunodeficiency virus type 1 (HIV-1) (1–3). The World Health Organization (WHO) estimated that ~26 million people would be receiving ART by the end of 2020 (4). Highly active antiretroviral therapy (HAART) reduces the incidence of opportunistic infections (OI), the progression to AIDS, and the death of HIV-infected patients through the enhancement of CD4^+^ T cells and the effective suppression of HIV viral load (5–7). Despite this, recent studies show that immune restoration by ART in some patients goes the wrong way, involving detrimental pathogen-specific inflammatory responses. This is termed immune reconstitution inflammatory syndrome (IRIS) and leads to a deteriorated clinical presentation of infections or tumor-related infections (8–10). Approximately 10–38% of HIV patients who initiated ART experience IRIS, usually within the first 6 months among severely immune-compromised individuals (11–14).

IRIS is presented as an exaggerated immune response against previously diagnosed and successfully treated pathogens before ART (called paradoxical IRIS) or the unmasking of a silent infection before treatment is initiated (15, 16). Evidence indicated that IRIS can be triggered by several etiologies, including mycobacterium, viruses (varicella zoster, herpes simplex, and Kaposi's sarcoma), fungus, and intestinal and tissue parasites (17–20). The symptoms are therefore heterogeneous, and hence their severity is highly dependent on the underlying pathogen or illness involved. Different etiologies can occur at the same time in the form of co-infection, which thereby woefully complicates the diagnosis and management of IRIS, typically in the context of low-income countries (17, 18, 21, 22).

A large number of studies have investigated and identified factors associated with IRIS. These include a low CD4^+^ count before ART, the presence of different OIs during treatment initiation, and the short duration of ART initiation after OI treatments (23–26). However, there is considerable heterogeneity between studies despite a strong, consistent finding on low CD4 counts (23, 26–30). In addition, the studies were carried out using nearly similar criteria to define IRIS, indicating that the lack of specific diagnostic tests remains a major challenge for accurate case identification (16, 30, 31).

Many studies have shown that East Africa, a region hardly hit by the pandemic, and Africa as a whole have higher incidences and predictors of HIV-associated IRIS than other regions (28, 32, 33). Since most of the studies focused on a single pathogen and a particular country, it is essential to compile information regarding the magnitude and associated factors of IRIS in the context of Eastern Africa, which will have paramount importance for policymakers. Furthermore, while the global number of new HIV infections is declining, Southern and Eastern African regions represent 47% of the global infection (34), indicating more attention and studies are needed for appropriate management. Hence, the current review is aimed at estimating the magnitude of IRIS along with its effect on ART care and identifying the predictors and etiologies (infectious and non-infectious) of IRIS with regard to Eastern Africa.

## Methods

### Reporting and registration

The standard Preferred Reporting Items for Systematic Review and Meta-analysis (PRISMA) checklist was used to present the findings of the current study (43). The protocol has been registered in the International Prospective Register of Systematic Reviews (PROSPERO) with the registration number CRD4202016413.

### Search strategy

A comprehensive systematic search on four electronic databases, namely Google Scholar, PubMed, Web of Science, and free Google access was performed from the start of the study up to 5 June 2021, and the search was lastly updated on 30 June 2022. The exploration scheme applied to this review was Conditions, Context, and Population (CoCoPop). The search terms used were a combination of relevant Medical Subject Headings (MeSH) and database-specific terms. Search key terms, including “HIV,” “ART,” “HAART,” “Human Immune Deficiency Virus,” “Immune Reconstitution Inflammatory Syndrome,” “East Africa,” “Kenya,” “Tanzania,” “Uganda,” “Ethiopia,” “Rwanda,” “Somalia,” “Madagascar,” “Eritrea,” “Burundi,” “Mozambique,” “South Sudan,” “Sudan,” “Malawi,” “Zambia,” “Djibouti,” “Mauritius,” “Seychelles,” “Zimbabwe,” and “Comoros.” Boolean operator combinations (AND, OR) were used to optimize the search results.

### Eligibility criteria

Studies were considered eligible based on the following criteria: (1) published in peer-reviewed journals without restriction on the study designs (randomized controlled trials [RCT] or observational studies); (2) studies conducted in one of the East African countries mentioned above; (3) studies of individuals diagnosed with either paradoxical or unmasking IRIS and reported the incidence or risk factors or etiologies of HIV-associated IRIS.

### Study selection and quality appraisal

The removal of duplicates was performed using reference management software, such as EndNote X8. In the meantime, authors (HA, MK, and AG) independently screened the titles, abstracts, and later full texts based on predefined eligibility criteria. Any difference was reconciled by a third author (DM). The quality of the research articles was evaluated by two independent reviewers (SB and AG) according to the Joanna Brigg Institute's (JBI) quality appraisal criteria (44). Eleven studies (28, 33, 35–42, 45) were evaluated by the JBI checklist developed for each of the observational studies (cross-sectional, case-control, and cohort studies). The independent reviewers came together and resolved any discrepancies in scoring through agreement. Studies with an average score of 50% and above were included in this study.

### Data extraction

Two authors (AG and DM) extracted all the important data using a standardized Microsoft Excel spreadsheet. A third author (HA) was involved to resolve any of the discrepancies the two authors could not agree upon. The data extracted comprised the last name of the first author, years of publication, study place, IRIS prevalence with 95% CI, sample size, study design, types of IRIS, causes of IRIS, and possible factors related to IRIS.

### Outcomes of interest

The proportion of individuals who developed IRIS after ART, the causes, and the factors associated with the development of IRIS were the primary outcomes of interest in this review.

### Data analysis

The extracted data were exported to Stata version 17 for meta-analysis. The random-effects model has been in practice for pooled estimation of IRIS among HIV patients, given the considerable heterogeneity between the primary studies (I^2^ = 95.6%, *p* < 0.001) (44). The existence of publication bias was evaluated by looking at the symmetry of the funnel plot, and by determining with a *p*-value of < 0.05 in Egger's test if there was a considerable publication bias. The study country was used for sub-group analyses of the heterogeneous studies included (46).

## Results

### Search results

**Figure 1:**
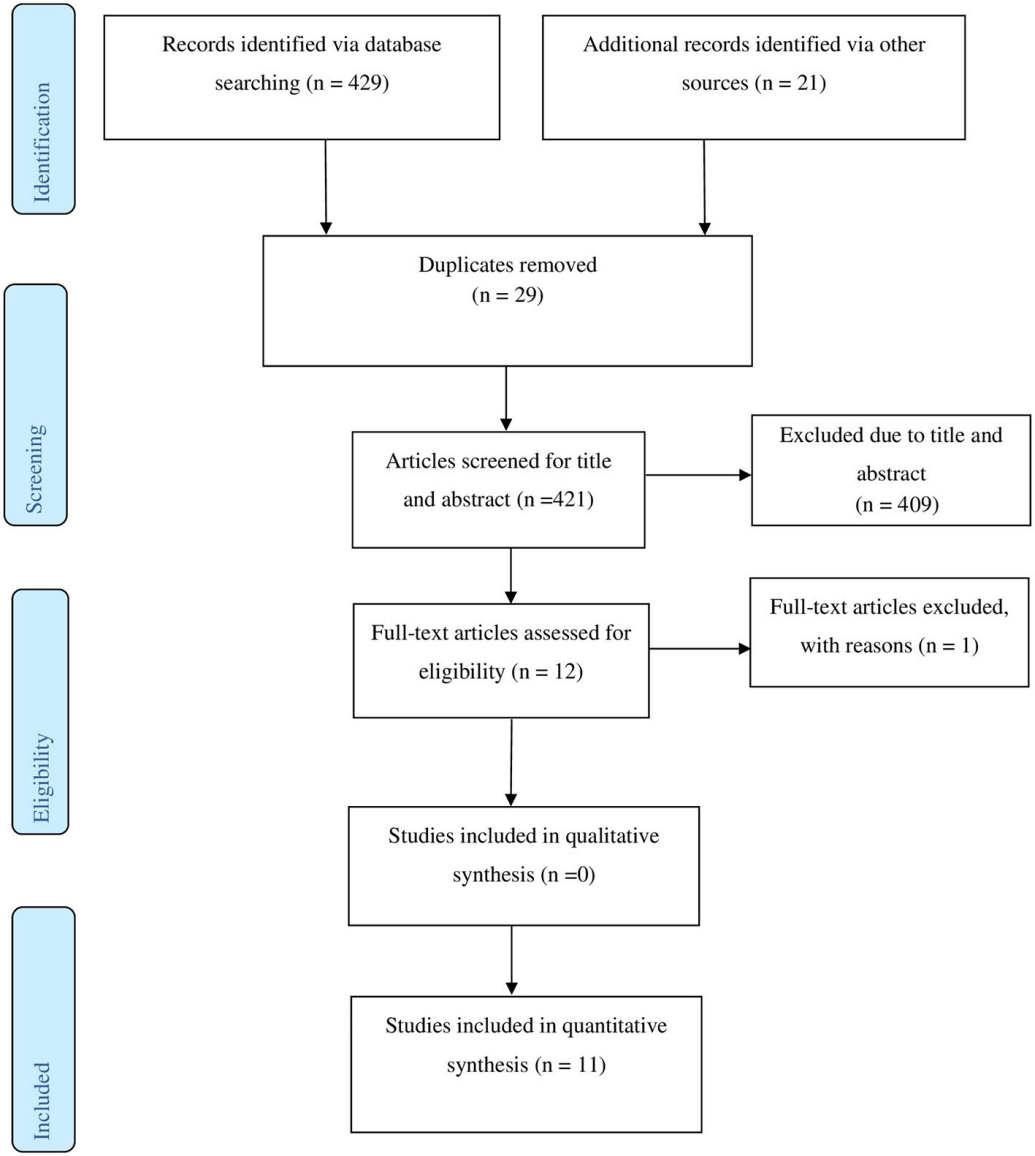
PRISMA flowchart.

Our systematic search from different databases identified 450 potential articles. After duplicates were eliminated, 421 articles remained. Screening the 421 articles for title and abstract revealed the exclusion of 411 articles. Finally, 11 studies met our eligibility criteria and were included in the meta-analysis. A summary of the steps involved in the screening of articles is indicated in Figure 1.

### Characteristics of studies included

A total of 11 studies were selected. Three of the studies included were from Uganda (38–40), three from Ethiopia (28, 33, 35), three from Mozambique (36, 41, 45), one from Tanzania (37), and one from Kenya (42). There were eight prospective studies, one retrospective, one case-control, one chart review, and one clinical trial. All of the included studies were published from 2008 onward, with more than half (seven) published between 2010 and 2013. The latest article was published in 2017. A summary of the characteristics of the included studies is provided in Table 1.

**Table 1:** Characteristics of the included studies.

**Author(s) [year]**	**Country**	**Study area**	***N***	**Study design**	***p***
Huruy et al. (35)	Ethiopia	ZMH	186	Cohort	17.2
Klotz et al. (33)	Ethiopia	DRH	1,002	CS	7.39
Ali et al. (28)	Ethiopia	DRH	1,977	Case-control	7.23
Letang et al. (32)	Mozambique	MDH	136	Cohort	26.5
Bonnet et al. (36)	Mozambique	JMH, MH, AMHC	573	CC	9.25
Vanobberghen et al. (37)	Tanzania	IHI	7,010	Cohort	1.90
Worodria et al. (38)	Uganda	IDC	225	Cohort	1.33
Worodria et al. (39)	Uganda	MNTLP	258	Cohort	20.5
Orikiiriza et al. (40)	Uganda	JCRC	263	CS	38
Letang et al. (41)	Mozambique	MDH	69	Cohort	11.6
Ogola et al. (42)	Kenya	Uyoma	71	Case-control	36.6

N; sample size, P; proportion, ZMH; Zewditu Memorial Hospital, DRH; Dessie Referral Hospital, MDH; Manhica District Hospital, JMH; José Macamo Hospital, MH; Mavalane Hospital, AMHC; Alto Maé Health Center, IHI; Ifakara Health Institute, IDC; Infectious Diseases Clinic, MNTLP; Mulago National Tuberculosis and Leprosy Programme clinic, JCRC; Three Joint Clinical Research Center clinics, MDH; Manhicxa District Hospital, CS; cross-sectional, CC; clinical trial.

### Prevalence of HIV-associated IRIS

**Figure 2:**
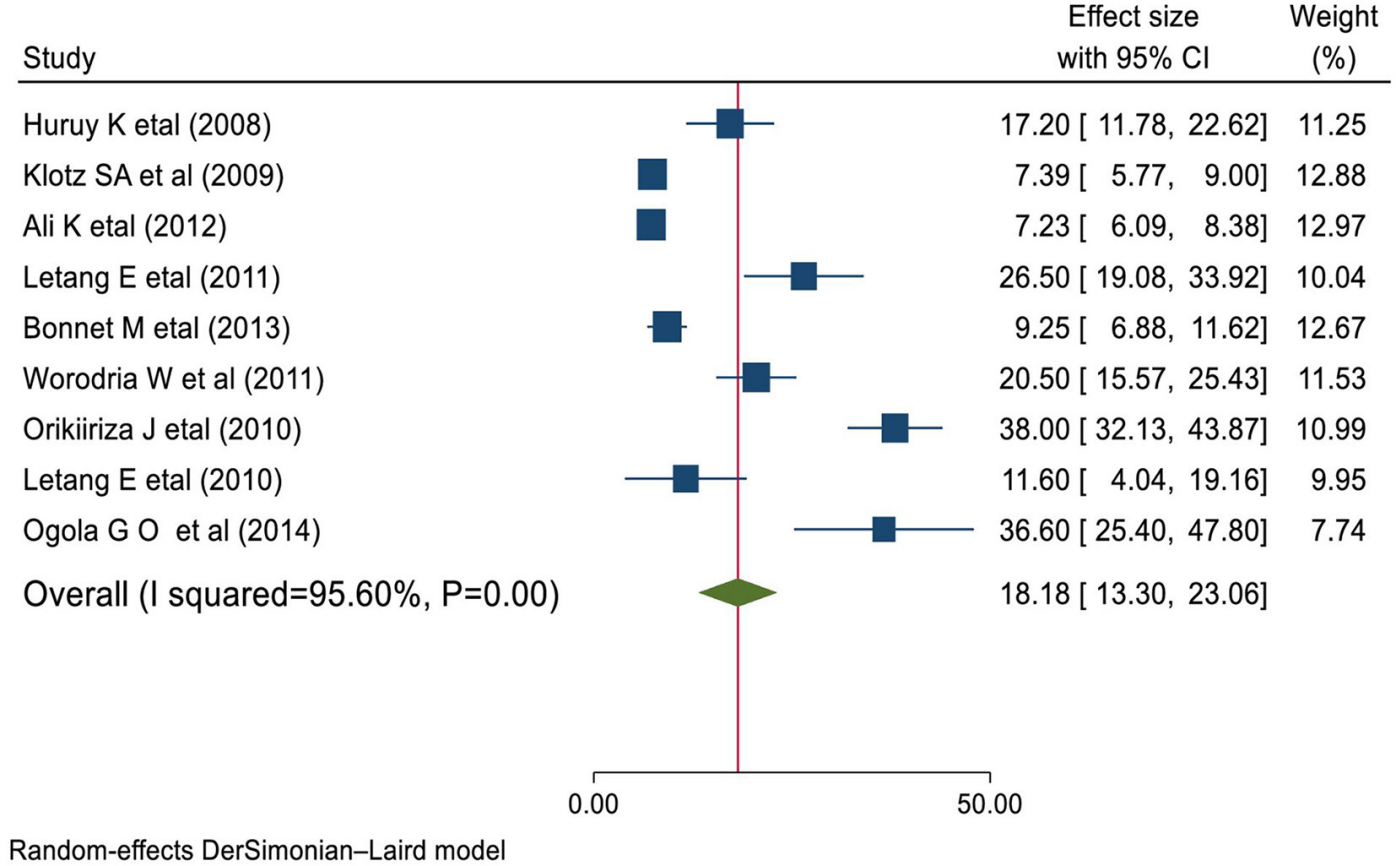
Forest plot of the prevalence of IRIS among HIV patients.

The studies used for this review included a total of 11,770 HIV-infected individuals, of whom the proportion of subjects with IRIS ranged from 7.2% (28) to 38% (45). The pooled prevalence of IRIS was 18.18% (95% CI 13.30–23.06), ranging from 7.2% to 38.0%. There was significant heterogeneity between the primary studies (*I*^2^ = 95.6%, *P* < 0.001) as indicated in Figure 2.

### Publication bias

**Figure 3:**
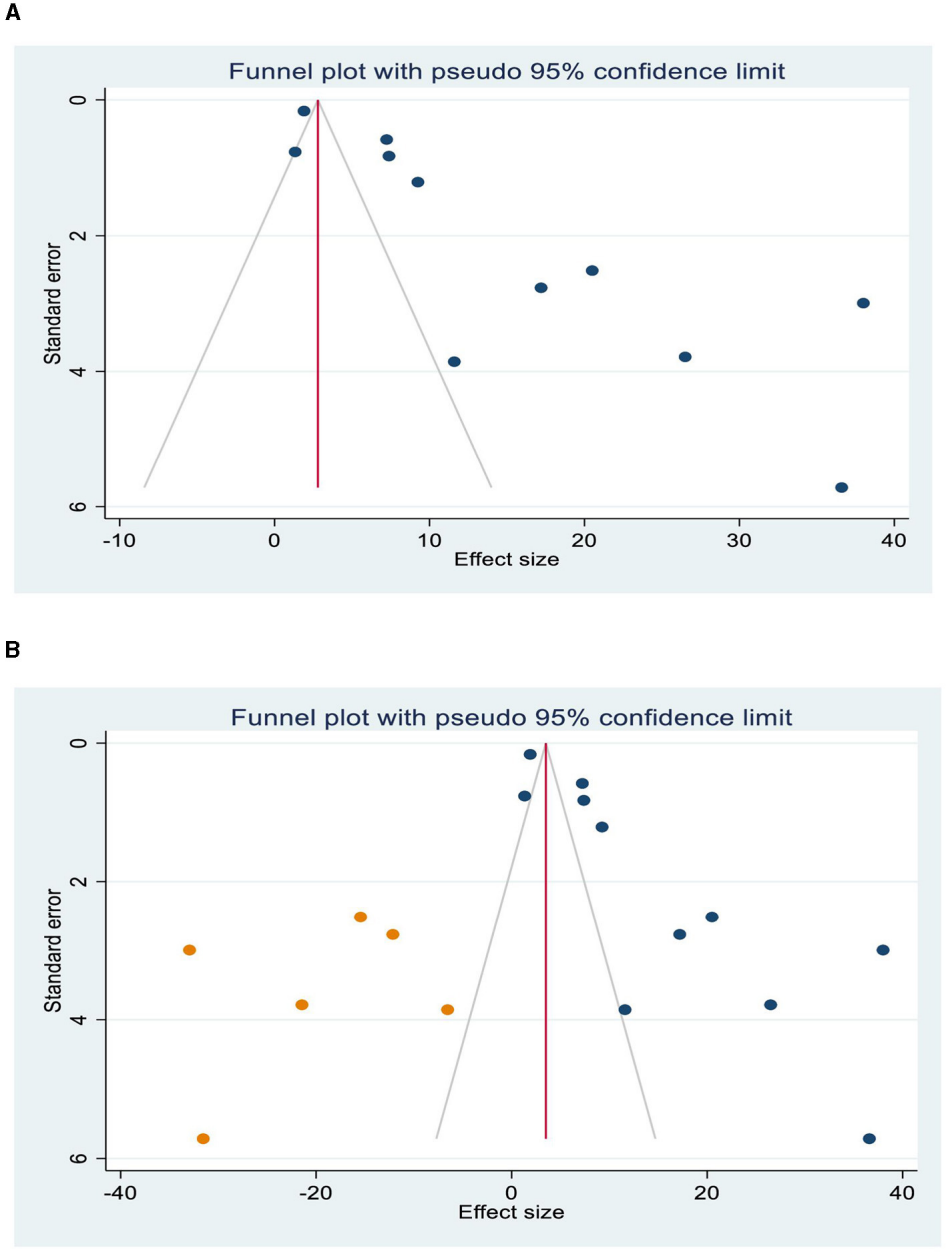
Publication bias, Funnel plots show the presence of publication bias **(A)**, and trim and fill analysis was used to resolve the bias **(B)**.

The asymmetrical distribution of the funnel plot has displayed the presence of publication bias among the primary studies included in the review (Figure 3A). The Egger's test result (*p* < 0.001) has also indicated a significant study effect bias of HIV-related IRIS. Therefore, to adjust the overall bias effect estimate, we conducted a trim and fill method analysis (Figure 3B).

### Investigation of heterogeneity

The percentage of *I*^2^ statistics in the forest plot shows substantial heterogeneity across the included studies (*I*^2^ = 95.6, *P* < 0.001). Thus, to minimize the heterogeneity, sensitivity analysis and sub-group analysis were performed.

### Sensitivity analysis

**Figure 4:**
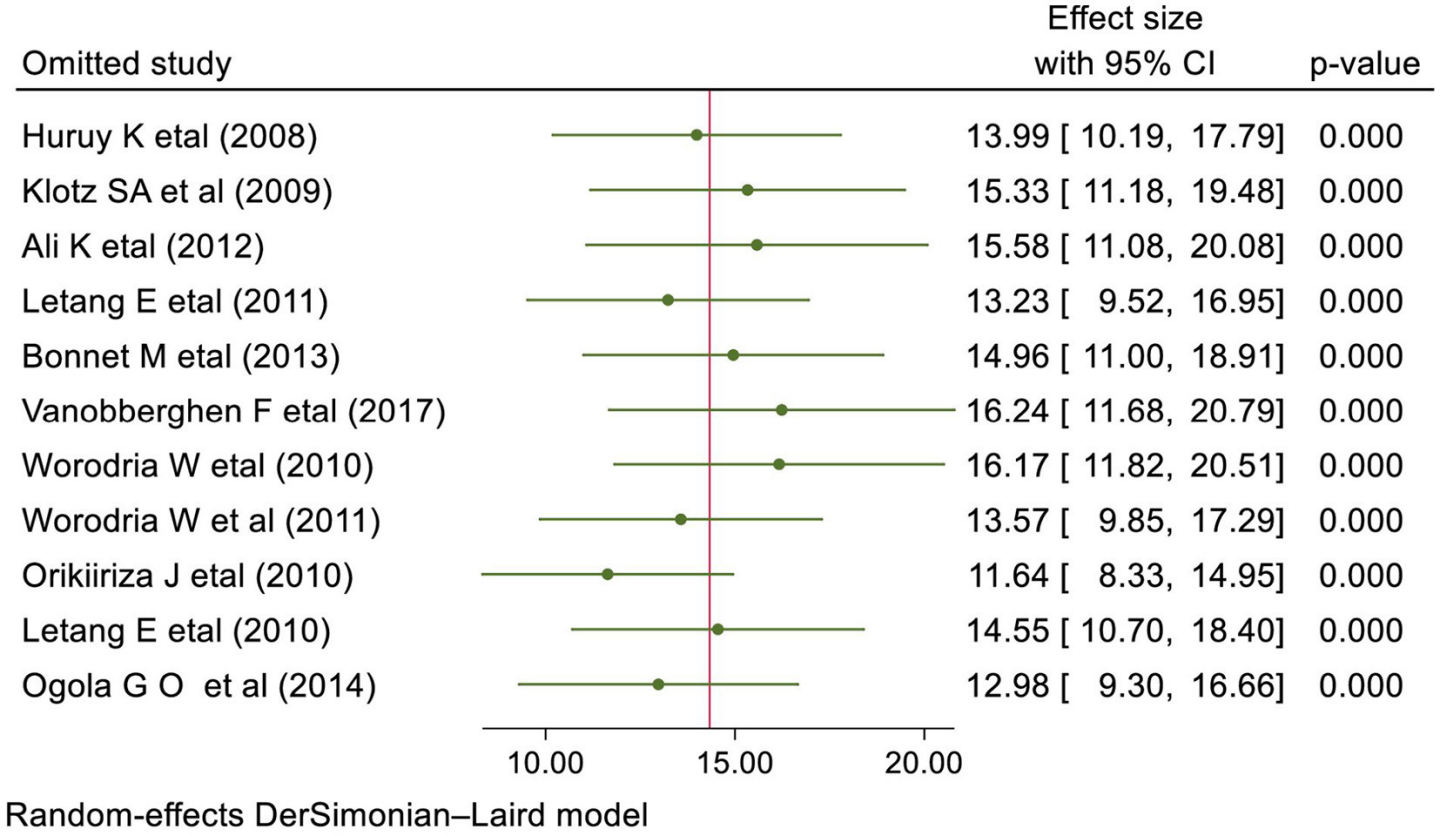
Sensitivity analysis presenting the influence of single study on the overall prevalence of IRIS among HIV-infected individuals.

The result of the sensitivity analysis presented in Figure 4 showed that the effect size of individual primary studies was moderately close to the overall pooled effect size, i.e., the effect size of each included study when omitted has a moderate effect on the pooled estimate.

### Subgroup analysis

**Figure 5:**
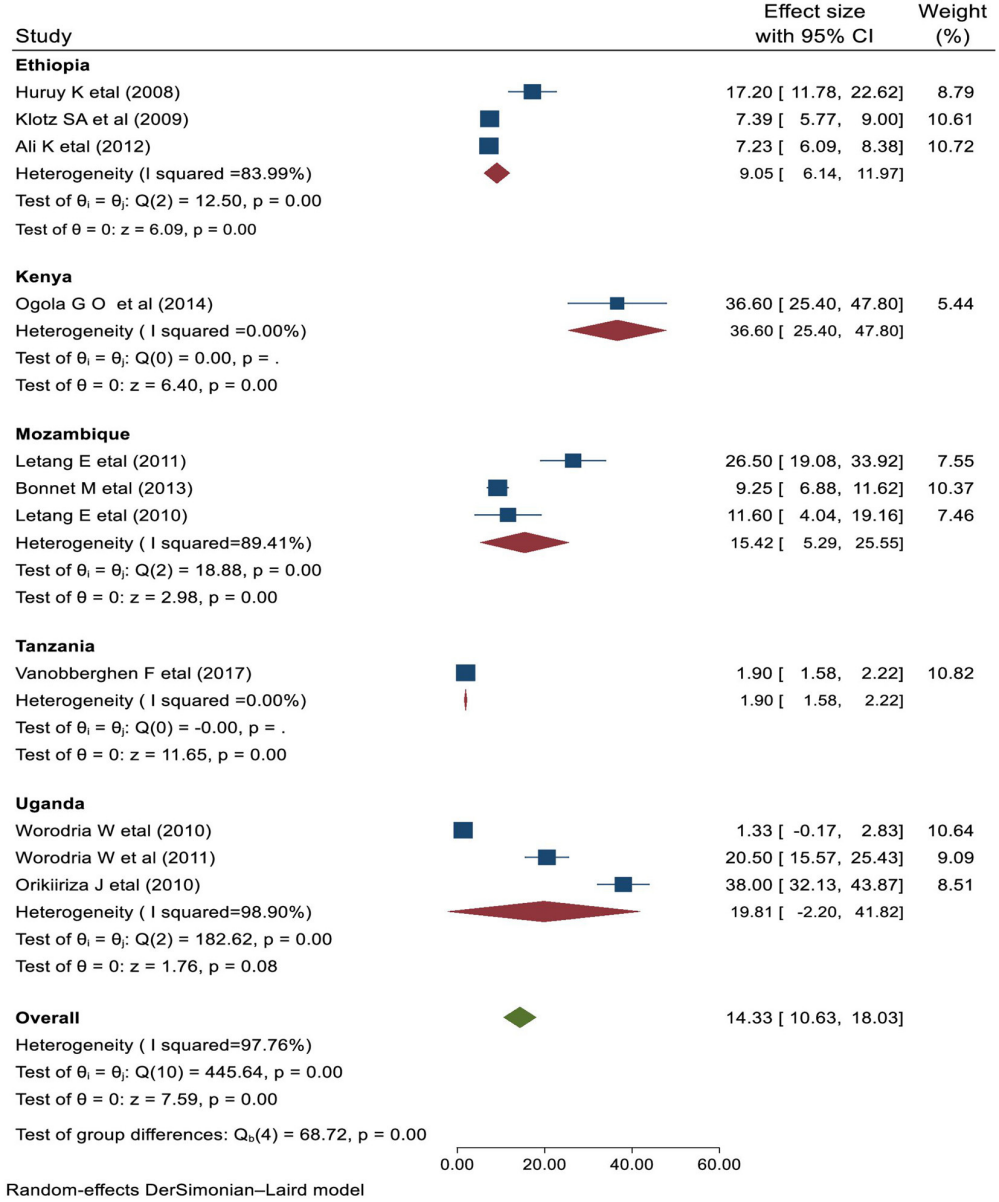
Sub-group analysis by the country of study on the prevalence of IRIS in HIV patients.

The country-based sub-group analysis has been estimated for Ethiopia, Mozambique, and Uganda. Among the included studies, three reported the prevalence of HIV-associated IRIS in Ethiopia, with a pooled prevalence of 9.05% (95% CI 6.14–11.97), three from Mozambique, with a pooled estimate of 15.43% (95% CI 5.29–25.37), and three from Uganda, with a pooled burden of 19.81% (95% CI −2.20 to 41.82) (Figure 5).

The studies included in this review were scrutinized to see if they followed standard case definitions. However, IRIS was characterized as paradoxical and unmasking in only four of the eleven studies (28, 39, 40, 45).

### Causes and factors associated with IRIS

**Figure 6:**
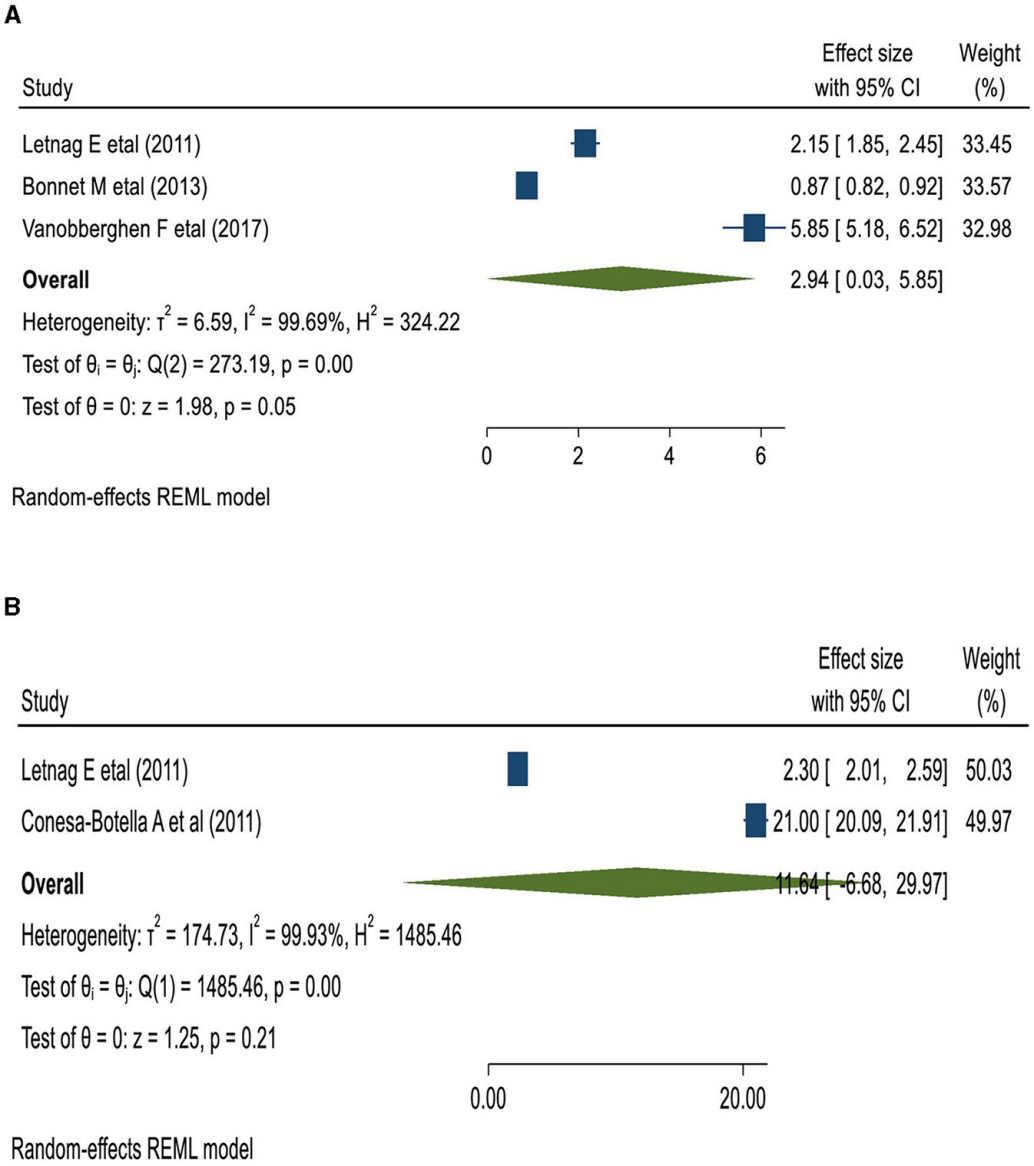
The pooled odds of IRIS among BMI < 18.5 kg/m^3^ **(A)**; and the lower pre-ART CD4 count patients in East Africa **(B)**.

Tuberculosis (33, 35, 38), herpes viruses (35), Cryptococcus (33, 35), Toxoplasma (33, 35), and Schistosoma (42) were the etiologies of opportunistic illnesses associated with IRIS. Underweight (body mass index [BMI] < 18.55 kg/m^2^) patients were 2.94 times more likely to develop IRIS than their normal-weight counterparts. Similarly, patients with a lower pre-ART CD4 count of < 50 cells/μl had an 11.64 times higher chance of developing IRIS (45) (Figure 6).

## Discussion

IRIS has been estimated to occur in 10–32% of HIV-infected patients beginning ART (47). In the eastern part of Africa (Ethiopia, Eritrea, Somalia, Djibouti, Sudan, Uganda, Tanzania, Kenya, Mozambique, Malawi, Rwanda, Burundi, and Madagascar) with a higher HIV hit, the development of IRIS was up to 38% (35, 40, 42, 45). Owing to the diagnostic challenges in Africa, many of the studies included did not adhere to standards, including the classification of IRIS. Only few of the studies included in this meta-analysis classify IRIS as paradoxical and unmasking IRIS (28, 39, 40, 45).

As different studies in Eastern African countries showed inconsistencies in the burden of HIV-associated IRIS, we conducted this review to assess its overall burden and impact on ART care (39, 40). The pooled prevalence of IRIS among the eleven East African studies comprising 11,770 HIV-infected individuals was 18.18% with a higher variation in geographic location. This could have reflected the differences in rates of late diagnosis, diagnostic criteria used, or prevalence of associated OI (33, 35, 37, 39, 40, 45). The higher magnitude of IRIS in the region is implicated in poor adherence and compliance with ART, the increased risk of resistance to ART medications, and the significant morbidity and mortality of people living with HIV/AIDS (48, 49). Importantly, people with IRIS have a mortality risk that is more than twice as high as that of those without (48).

People infected with HIV in low- or middle-income countries (LMICs) are likely to start ART with severe immune system impairment and a low CD4+ T-cell count. The primary reason for this could be delayed diagnosis and late presentation to HIV care and treatment (50, 51). A low CD4+ T-cell count increases the risk of OIs, and when ART is initiated, the immune response to an active (but also sub-clinical) opportunistic agent worsens the clinical condition (51). The current review indicated that a lower pre-ART CD4 T-cell count of < 50 cells/μl is strongly linked to IRIS development. In line with this report, a meta-analysis of data from 22 cohort studies showed that the incidence of IRIS increases exponentially as the CD4 count decreases (11). Starting ART at a younger age, CD4 T-cell count of < 100 cells/μl, an accelerating rise in CD4 count immediately after ART, the presence of disseminated OIs at the start of ART, and a rapid decline in viral load are considered among the commonly identified risk factors for IRIS (47, 49, 52). Our review also indicated that being underweight (BMI < 18.5 kg/m^2^) is associated with the development of IRIS. A low baseline BMI level was reported as one of the useful predictors of IRIS and its associated death (53). A large prospective international cohort study also reported that there were low BMI levels among patients with viral-associated IRIS than among non-IRIS patients (54).

Immune reconstitution in HIV patients has been linked to OIs and autoimmune disorders or immune-mediated inflammatory diseases (52, 55). Our review shows OIs such as tuberculosis, herpes, Cryptococcosis, toxoplasmosis, and schistosomiasis have been linked to the infectious IRIS in East Africa. A variety of fungal (e.g., Cryptococcus, Pneumocystis, Histoplasma, and Candida), viral (e.g., herpes, cytomegalovirus, hepatitis viruses, and John Cunningham virus), bacterial (e.g., Mycobacteria and Bartonella), and parasitic (e.g., Toxoplasma, Leishmania, Schistosoma, and Cryptosporidia) OIs can cause latent or sub-acute infections in HIV/AIDS patients and are associated with IRIS (47, 49).

The underlying antigenic burden (from both viable and nonviable opportunistic pathogens), the degree of immune restoration following ART (e.g., the level of change in the CD4+ T-cell count), and host susceptibility may all play a role in IRIS pathogenesis (14). Knowing about these factors can help clinicians decide when to start ART or use prophylactic measures. There are different reports worldwide focusing on the predictors of pathogen-associated IRIS, and there are ample data on this subject. For instance, persistent Cerebrospinal fluid cryptococcal growth at ART initiation, pre-ART increases in Th-cell responses, high pre-ART plasma IL-5 and IL-7 levels, and a lack of pro-inflammatory cytokine responses were all mentioned as strong predictors of Cryptococcus-associated IRIS (56–58). Higher expressions of inflammatory markers such as IL-17 and IL-6 were reported as predictors of TB-associated IRIS (54). A positive urinary TB lipoarabinomannan was also reported as a predictor of TB-associated IRIS (59). More importantly, >10% weight loss (25), low hemoglobin levels (25, 35, 37), increased liver function enzymes (aspartate aminotransferase [AST] and alanine aminotransferase [ALT] levels) (35, 37), and increased serum C-reactive protein [CRP] (≥5 mg/l) (25, 37, 39) were identified as potential biomarkers for OI-associated IRIS, particularly for mycobacterial-associated IRIS, in developing countries. There was significant heterogeneity between the studies used in this review. Factors such as differences in IRIS diagnostic criteria or definition, the nature of the study (controlled or uncontrolled), the CD4 T-cell count at the start of ART, and differences in study populations with differing risk profiles may have contributed to between-study heterogeneity (11, 47, 51).

## Conclusion

The controversial burden of IRIS was 18.18% in the Eastern African region. The lower pre-ART CD4 T-cell count of < 50 cells/μl and the low baseline BMI level were important predictors of IRIS in this region. However, easily accessible laboratory tests such as hemoglobin, AST and ALT, serum CRP, and significant weight loss are potential markers for OI-associated IRIS to be considered in resource-limited settings. Viruses, bacteria, fungi, and parasites were the etiologies of IRIS, indicating the need for a deep understanding of the pathogen-specific immune pathogenesis of IRIS for targeted therapies. Because of the pathogen-specific case definitions, adherence to standard case definitions might be difficult to follow. Overall, it is imperative to focus on the early identification and management of HIV-associated IRIS to prevent IRIS-related morbidity and mortality.

## Data availability statement

The original contributions presented in the study are included in the article/supplementary material, further inquiries can be directed to the corresponding author.

## Author contributions

All authors listed have made a substantial, direct, and intellectual contribution to the work and approved it for publication.
